# “We are supposed to take care of it”: a qualitative examination of care and repair behaviour of long-lasting, insecticide-treated nets in Nasarawa State, Nigeria

**DOI:** 10.1186/1475-2875-13-320

**Published:** 2014-08-14

**Authors:** Gabrielle C Hunter, Leah Scandurra, Angela Acosta, Hannah Koenker, Emmanuel Obi, Rachel Weber

**Affiliations:** Johns Hopkins Center for Communication Programs, 111 Market Place, Ste 310, 21202 Baltimore, MD USA; Malaria Consortium, 3rd Floor, Abia House, Central Business District, Abuja, Nigeria

**Keywords:** Long-lasting insecticidal nets, Care, Repair, Qualitative, Nigeria, Mosquito net, Behaviour, Bed net, Insecticide treated net

## Abstract

**Background:**

The longevity of long-lasting insecticidal nets (LLIN) under field conditions has important implications for malaria vector control. The behaviour of bed net users, including net care and repair, may protect or damage bed nets and impact the physical integrity of nets. However, this behaviour, and the motivating and inhibiting factors, is not well understood.

**Methods:**

Qualitative research methods were used to examine behaviour, attitudes and norms around damage, care and repair of LLINs. Eighteen in-depth interviews (IDI) and six focus group discussions (FGD) were conducted with LLIN users in two local government areas of Nasarawa State, Nigeria. A brief background questionnaire with the 73 participants prior to IDIs or FGDs collected additional data on demographics, net use, and care and repair behaviour.

**Results:**

Respondents cited that the major causes of damage to bed nets are primarily children, followed by rodents, everyday handling that is not gentle, and characteristics of sleeping spaces. Caring for nets was perceived as both preventing damage by careful handling and keeping the net clean, which may lead to over-washing of LLINs. Repairing a damaged net was considered something that net users should do and the responsibility of adults in the household. Despite this, reported frequency of net repair was low (18%). Motivations for taking care of and repairing nets centred around caring for one’s family, avoiding mosquito bites, saving money, and maintaining the positive opinion of others by keeping a clean and intact net. Barriers to net care and repair related to time availability and low perceived value of bed nets or of one’s health.

**Conclusion:**

This study provides novel and valuable insights on the perceptions and attitudes of LLIN users in Nasarawa, Nigeria on the durability of bed nets, how to care for and repair nets, and for what reasons. Communication around net care should stress proper daily storage of nets, regular net inspections, prompt repairs, and clarify misconceptions about proper washing frequency and technique. These messages should include compelling motivators, such as local social norms of household hygiene.

## Background

Long-lasting, insecticide-treated bed nets (LLINs) are the cornerstone of global malaria prevention and control. A systematic review of numerous studies has demonstrated LLIN effectiveness in reducing malaria morbidity and mortality in vulnerable populations [1]. Despite this, the effective lifespan of nets under field conditions, or net durability, is still largely unknown [2, 3]. Net durability has critical implications for malaria transmission since nets in poor or degraded condition may be less protective against malaria [2, 4, 5]. Although the effect of net damage on malaria parasitaemia may be unclear, if damaged nets lead to user-determined end of use, they cannot protect against malaria. Indeed, a net user’s decision to discontinue use of a bed net may be related to physical integrity [6–11]. Further, understanding net durability is important as funding for LLINs becomes increasingly tenuous in the foreseeable future [12] and donors and governments look for ways of prolonging net lifespan for greater value for money.

Net durability is defined by the World Health Organization (WHO) using three categories: 1) net survivorship and net attrition, 2) fabric integrity, and, 3) insecticidal activity (bio-efficacy). The components of durability are determined by both intrinsic factors related to the manufacture of the net (e g, composition, knitting pattern, insecticide type and content) and extrinsic factors that cause damage and degradation [3]. Currently, the majority of LLINs distributed throughout sub-Saharan Africa are designed to retain adequate amounts of insecticide to last for up to 20 washes per the WHO definition of an LLIN [13]. Budgeting for LLIN distribution to replace worn nets is generally based on an assumed average lifespan estimate of three to five years, with net replacement recommended every three years [3]. However, recent studies suggest that the assumed lifespan may be over-estimated and that nets may reach compromised or degraded conditions much faster in field conditions, with LLIN durability influenced by household composition, socio-economic conditions, or climatic settings [9, 14–17].

The behaviour of net users, such as net care and repair practices, may also affect the duration of the useful life of nets. The authors conceptualize behaviour intended for prolonging net life at the household level into two areas: net care and net repair. Net care is defined here as any net prolonging behaviour carried out at the household level to prevent net damage. Net care behaviour includes handling and storing a net carefully, especially during the day and other times when the bed net is not in use. Net repair is defined here as any behaviour carried out at the household level to close holes and tears in bed nets. Net repair behaviour can be carried out in multiple ways, including closing a hole by sewing, by tying using a knot, string or elastic band, or by patching.

There are mixed research findings on the relationship between the protective lifespan of LLINs and net care and repair behaviour, including the frequency and technique of washing LLINs [9, 16, 18–21]. However, little is known about this behaviour and, particularly from a qualitative point of view, what net care and repair activities are practiced, how this behaviour is perceived by net users, and how to promote certain behaviour that may extend the useful life of nets. Behaviour change communication interventions for malaria prevention may include net care messaging, but do not usually include messages on how and why to repair nets. The current version of the Alliance for Malaria Prevention toolkit [22], an important source of recommendations for LLIN distribution campaigns, only briefly mentions net care and repair messaging in its BCC section, with a short list of net care and repair behaviors that a program might consider promoting after LLIN distribution campaigns.

Despite growing interest in the role that net care and repair may play in durability, there are barriers to this behaviour; net care is not always straightforward nor is it perceived to be important. A 2006 study in The Gambia showed that, while people used their nets regularly, these nets often went unrepaired due to the competing demands of household tasks and livelihood responsibilities [18]. A study in Ethiopia showed that the overriding reasons for not using nets were that they were too torn (46%); however, only 4% of users had made net repairs in the three years following a mass distribution and 32% of nets had been discarded [6]. A study in Laos showed that despite maintenance instructions printed on labels on LLINs, about 40% of nets observed after two to three years of use had holes, were torn and the text-only maintenance instructions had not been followed sufficiently [16].

Other experiences have indicated that strategic behaviour-change interventions may affect understanding of net care and repair behaviour. In Tanzania, knowledge about the importance of repairing bed nets started at only 37.1% but increased to 90.4% following educational sessions on malaria-related topics, including net repair, and was sustained in follow-up 15 months later [11]. In The Gambia, a behaviour-change intervention involving posters and locally composed songs to promote net care and repair was associated with an increase in net repairs, finding an increase from 27 to 41% of holes repaired on average per net over a four-month period [18].

Still, there remains a gap in both the quantitative and qualitative research examining the specific behaviour, motivators and barriers to net care and repair and how these factors may influence the lifespan and protective efficacy of nets. This paper examines net care and repair behaviour in Nasarawa State, Nigeria from a qualitative perspective and sheds light on the knowledge, attitudes and practices about net care and repair based on research in two local government areas (LGAs), with particular attention to the perceived barriers and motivators to net care and repair behaviour. The results from this formative research were used to guide a behaviour-change communication (BCC) intervention in this setting. Although the results presented here are specific to Nigeria, some findings may be applicable and relevant in other contexts and may inform net use communication campaigns in a variety of settings.

## Methods

### Study setting

**Figure 1:**
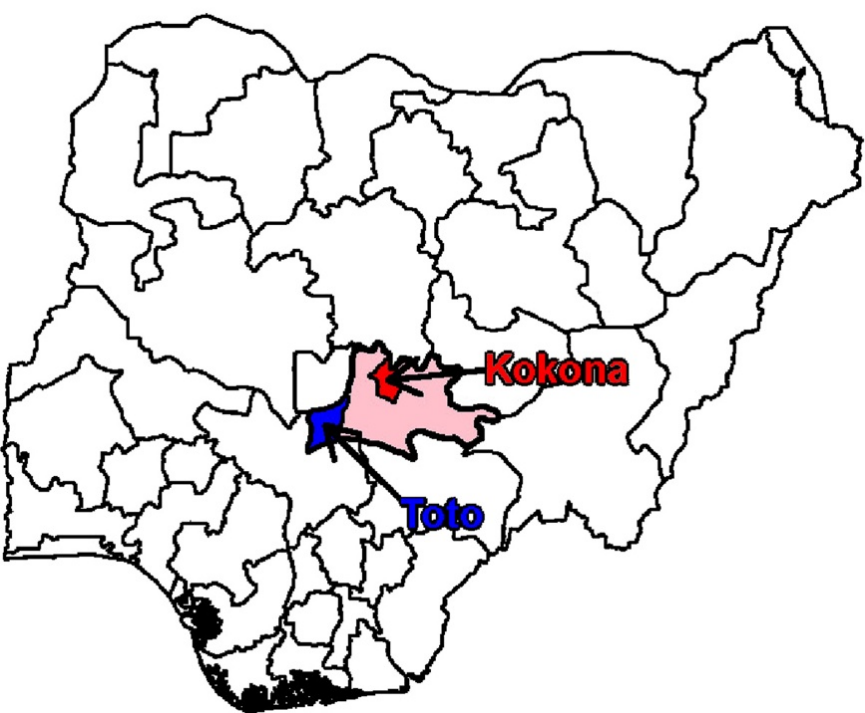
**Location of study sites (Kokona and Toto) within Nasarawa state, Nigeria.** Pink shading indicates Nasarawa state. Red shading indicates the location of Kokona LGA (site of future intervention). Blue shading indicates the location of Toto LGA (control site). Data were collected from both sites for this qualitative study.

This qualitative study was designed to inform a BCC campaign that would take place in 20 rural and peri-urban settlements of one of the 13 LGAs of Nasarawa State (Figure 1). In addition, a baseline and endline evaluation before and after the campaign would compare outcomes between the intervention LGA and a control LGA. Due to the primary intention to inform intervention development, two-thirds of the data were collected from the intervention LGA (Kokona), and one-third from the control LGA (Toto) for comparison. In each of the two LGAs, data were collected from one rural and one peri-urban locality, for a total of four study sites.

The first campaign to distribute LLINs targeting the general population took place in Nasarawa state in 2009–2010, two years before this study, and nets were completely subsidized. Prior to this campaign, LLINs had been targeted to pregnant women and young children, also free of cost, but were not available through any other source in significant numbers. Before the mass campaign net coverage in the state was measured at 13.8% of households owning at least one ITN. One year after the campaign of 2009–2010 net coverage increased to 62.5% of households owning at least one ITN. This same survey also found that net use was strong in households; of households with access to an ITN, 77% used it [23]. At the time of this study, no additional campaigns had been conducted to replace nets, and participant experiences with net care and repair would mostly have been in relation to the two-year old campaign nets.

### Study design

Qualitative methods were used to gain an in-depth understanding of the knowledge, attitudes, practices, perceived barriers, and motivations regarding net care and repair behaviour. Interviews were conducted in Igbira and Hausa languages by a data collection team fluent in these two local languages. Interview and focus group guides were pre-tested on-site in Nigeria.

In each study site, permission to carry out this study was sought from the chief of the LGA or the village head. In each site, a starting household was defined, and one adult net-user per household recruited systematically from every third household without replacement. Table 1 displays the breakdown of participants in the different study activities. A total of 18 in-depth interviews (IDIs) were conducted with the following three participant groups: adult men with nets hung over their sleeping spaces the previous night (n = 7), adult women with nets hung over their sleeping spaces the previous night (n = 5), and mothers of children under five years old who had at least one net hung the previous night in the household (n = 6). Six single-sex focus group discussions (FGDs) were also conducted with male and female caretakers of children under ten years old who had at least one net hung over a sleeping space in the household the previous night. Seven to ten persons participated in each FGD. FGDs were conducted in a community location and IDIs were conducted in participants’ homes. Ethical clearance was obtained for conducting human subject research from the Johns Hopkins Bloomberg School of Public Health Institutional Review Board, as well as from the National Health Research Ethics Committee of Nigeria. All participants provided written informed consent before beginning data collection.

**Table 1:** **Distribution of study participants in focus group discussions and in-depth interviews across Kokona and Toto local government areas**

Study activity	Type of participant	Kokona LGA	Toto LGA	Total
**FGDs** (N = 6)	Men	2	1	3 FGDs
	Women	2	1	3 FGDs
**IDIs** (N = 18)	Men	5	2	7 IDIs
	Women	2	3	5 IDIs
	Women with child <5	4	2	6 IDIs

### Data collection

All IDIs and FGDs were audio recorded, transcribed verbatim and translated into English. Semi-structured discussion guides were used during the FGDs and IDIs. During both IDIs and FGDs participants were asked about their experiences with the physical condition of nets, including how nets become damaged, how damage can be prevented, how nets can be repaired, the availability of materials to repair nets, what makes a net look ‘good’, and how and when nets are washed. Probes specifically addressed barriers to repairing nets, and the cost and time implications of net repair *versus* the cost of buying a new net. In addition, participants were shown a series of three nets in different conditions (good, some holes, many holes) and were asked what they thought about each net. Finally, questions were asked about norms regarding the appearance of nets (i e, torn, dirty, clean, intact) and how this would reflect on others’ opinions of those net users. All participants completed a one-page background questionnaire that captured household demographics, net use and whether they had ever washed or repaired a net.

### Data analysis

Data analysis was conducted using a codebook that was created during a group discussion on the main themes and sub-themes. Main themes were identified and all transcripts were then coded, by three members of the study team, according to the codebook. The study team used both pre-determined codes, following the main topic areas included in the IDI and FGD guides (inductive coding), and emergent codes to capture new themes that arose during analysis (deductive coding). Each coder populated an Excel matrix with quotes identified as relevant to each main theme and sub-theme. After coding all of the transcripts, the team reconvened several times to discuss findings, and rework main themes or sub-themes as necessary.

## Results

The brief background questionnaire provided information on participant demographics and reported practices on net use, care and repair. The qualitative focus groups and interviews yielded data on causes of net damage, barriers and motivators to net care and repair, as well as roles and responsibilities for net care and repair. Qualitative and quantitative data from the two sites were not different and therefore grouped together for analysis.

### Participant characteristics

Table 2 summarizes participant demographic characteristics in the IDIs and FGDs. Study participants included 37 men aged 25–70 years and 36 women aged 20–45 years and all participants had at least one child. The proportion of women to men participating in the IDIs was higher than in the FGDs because an additional category of women with children under five years of age was recruited for this activity. Table 3 lists net use and maintenance behaviour reported by study participants on the participant questionnaire. Only 18% of respondents reported having ever repaired a net although they were much more likely to have ever washed a net (65%). Women were more likely than men to have ever repaired a net (22.2 *versus* 13.5%) and about equally likely to have washed a net (69.4 *versus* 67.6%), although this study was not powered for statistical significance. The 73 participants used nets in their households, with 73.0% of men and 64.5% of women reporting sleeping under a net every night or most nights.

**Table 2:** **Participant demographic characteristics**

Demographic characteristics of participants	FGD	IDI	Total
	N = 55	N = 18	N = 73
**LGA of residence**	**N (%)**	**N (%)**	**N (%)**
Kokona	38 (69)	11 (61)	49 (67)
Toto	17 (31)	7 (39)	24 (33)
**Sex**	**N(%)**	**N(%)**	**N(%)**
Male	30 (55)	7 (39)	37 (51)
Female	25 (45)	11 (61)	36 (49)
**Median age**	35	27	33
**Marital status**	**N (%)**	**N (%)**	**N (%)**
Never married	1 (2)	2 (11)	3 (4)
Married	51 (93)	16 (89)	67 (92)
Widowed	3 (5)	0 (0)	3 (4)
Divorced	0 (0)	0 (0)	0
**Mean number of children**	6.2	4.8	5.8
**Mean household size**	12.8	11.5	12.5
**Mean number of nets in household**	5.1	3.8	4.8

**Table 3:** **Participant reported net use and maintenance behaviour**

Net use and maintenance behaviour	FGD	IDI	Total
	N = 55	N = 18	N = 63
**How often do you sleep under a net?**	**N (%)**	**N (%)**	**N (%)**
Never	0 (0)	0 (0)	0 (0)
Rarely	8 (15)	7 (39)	15 (21)
Occasionally	6 (11)	2 (11)	8 (11)
Most nights	9 (16)	3 (17)	12 (16)
Every night	32 (58)	6 (33)	38 (52)
**How often does your child sleep under a net?**	**N (%)**	**N (%)**	**N (%)**
Never	1 (2)	0 (0)	1 (1)
Rarely	9 (16)	1 (2)	10 (14)
Occasionally	7 (13)	2 (11)	9 (12)
Most nights	7 (13)	4 (22)	11 (15)
Every night	31 (56)	11 (61)	42 (58)
**Kind of sleeping spaces covered by nets**	**N (%)**	**N (%)**	**N (%)**
Mat on the floor	0 (0)	0 (0)	0 (0)
Mattress on the floor	22 (40)	7 (39)	29 (40)
Bed	33 (60)	11 (61)	44 (60)
Other	0 (0)	0 (0)	0 (0)
**Ever washed a net**	**N (%)**	**N (%)**	**N (%)**
No	16 (29)	7 (39)	23 (32)
Yes	39 (71)	11 (61)	50 (68)
**Ever repaired a net**	**N (%)**	**N (%)**	**N (%)**
No	44 (80)	16 (89)	60 (82)
Yes	11 (20)	2 (11)	13 (18)

### How nets become damaged

Of all topics discussed in this qualitative research, the cause of damage to nets was the most salient to participants and elicited richer description. Participants attributed damage of nets primarily to children, who reportedly caused holes when playing near nets.

*Some children can be very foolish in their playing; they can raise up any sharp object, and damage the net, knowingly or unknowingly to them*. Female with child aged under five years, Interview participant, Rural (Toto)

Other commonly cited sources of damage included rodents, pests and cockroaches. Soiling a net with food or rubbing hands on a net after eating were perceived to attract rats to damage nets. Aspects of everyday use and care such as storing, washing, and hanging also damaged nets. ‘Hanging’ a net referred both to the act of establishing the net’s hanging place in the house, as well as to the act of spreading out the net in the evening for use after having temporarily stored it during the day. In the former case, respondents mentioned that the nail or sticks used to hang a net can cause damage. Finally, certain characteristics of sleeping spaces were perceived to accelerate or facilitate damage to nets. These included using nets against walls or over mats, where the net consistently rubbed against the floor.

*Mostly the sleeping mat [damages the net]… It [a mat] is very hard for a mosquito net. It is not like soft mattresses some people use to have and not everybody can afford mattress.* Male, Interview participant, Rural (Kokona)

### Caring for nets

The concept of caring for a net was generally understood by participants as measures taken to prevent damage to nets, and were directly related to the causes of damage to nets. Participants cared for nets by preventing damage caused by children and rodents, practicing careful hanging and storage, and washing the net.

*Don’t clean your hand with it after eating to avoid damage from rodents.* Female, Focus group participant, Rural (Kokona)

Most commonly, participants stated that when not in use, they stored the net away from the reach of both children and rats, and discouraged soiling of the net with food to avoid attracting rats. Participants discussed the importance of putting away the net immediately upon waking up in the mornings by folding it or tying it up, and mentioned using care when arranging the net over the sleeping space in the evenings.

*The only way to prevent a hole [in a net] should be after use [of the net]; in the morning, it must be folded and kept safe. … I will immediately fold [the net] up and keep [it folded] after we have woken up*. Female, Interview participant, Peri-urban (Toto)

The majority of participants made reference to washing nets as an important part of caring for nets. However, there was no consensus among participants regarding how often to wash nets, and most stated they wash when it is ‘dirty’. Some mentioned washing their net weekly or bi-weekly, a frequency that would threaten net longevity. Methods for washing, including the type of soap to use, were also varied. A dirty net was not considered acceptable to some respondents and others noted that washing nets frequently had the advantage of keeping rodents away. In the context of washing and caring, several respondents also mentioned retreating the net with insecticide, and others understood that washing affects the insecticide concentration.

*I will not allow it to be dirty, if it is dirty I will wash it, if the net is always clean mosquito will not even come closer to it. Maintaining the net is very good, and whenever the chemical [is] finished you apply another one.* Female with child aged under five years, Interview participant, Peri-urban (Kokona)

A small number of participants mentioned other prevention measures, such as following care instructions given by providers of the nets, immediately sewing holes at the first sign of damage to keep them from getting larger, and daily inspections of net condition. The prevention behaviour was mentioned less frequently than keeping nets away from children and rodents, washing, and careful handling of nets.

### Repairing nets

Per the structured participant questionnaire, most respondents had little personal experience with net repair, with only 18% of respondents reporting having ever repaired a net themselves. However, in focus groups and interviews, nearly all respondents mentioned sewing, either in the home or by a tailor, as a method to repair those nets. When presented with examples of damaged nets, respondents discussed either their own experiences with net repair, what they would do to repair the example nets, or a combination of both. They also discussed their perceptions of how others in their home or community repair nets. Most participants, men and women alike, stated that they would take a net to be repaired by a tailor, but many also admitted that one can repair a net oneself. Materials needed to repair a net were a needle and thread, and most participants stated that both items are easily found in the market and are affordable for families, with prices ranging from NGN 50–80 (US$0.30-0.49). The cost of a tailor to mend a net was estimated around NGN 100 (US$0.61).

In the case when a patch was needed to repair a net, respondents largely stated that they would look for a piece of cloth that is similar to the net, either in texture, colour or both. It was common to suggest cutting patches from an old (‘condemned’) net that was no longer usable to repair a newer usable net.

*I will look for another torn net then cut the good part of the net and join it with this [torn] one and manage it.* Female, Focus group participant, Peri-urban (Kokona)

Participants stressed the importance of matching repair materials as closely as possible to the net, indicating a potential preference for maintaining a uniform appearance of nets close to their original state.

*You can also do the patching; a very white looking cloth material that may rather look like the net itself. Cut it according to the size of the damaged area of the net and [fix the net].* Female, Interview participant, Peri-urban (Toto)

### Family roles in caring, repairing and adapting nets

Responses about who in the family is responsible for net care and repair varied by the gender of the study participants, however most felt responsible for the net themselves. Female respondents most frequently stated that net care and repair is the job of the woman, wife and mother of the home, while male respondents most frequently cited that care and repair is the responsibility of the man, father and male head of household. However, some participants of either gender stated that net care and repair is the job of both males and females, and a smaller number of respondents nominated the opposite sex as the primary caretakers of nets.

Those who stated that net care is the man’s role felt that way because he is the head of the household and of the finances. It was perceived that the male head of household is responsible for preventing expenses due to malaria illness in his family, and that keeping nets intact could prevent such costs. Participants who cited that these tasks should be done by women described women as the caretakers of children and in charge of the housekeeping (setting the net out at night and storing it away in the morning was seen as a housekeeping task), and that men are too busy working outside of the home to take care of nets. There was consensus by respondents that children were considered unable to carry out the daily duties of caring for a net (hanging, arranging on sleeping space, folding in the morning, washing) with the necessary care to avoid damage. Both men and women mentioned parents as the persons responsible for net care and repair.

### Motivators to care and repair of nets

Respondents overwhelmingly expressed that net users should take care of their nets with one respondent summarizing a common point of view:

*We are supposed to take care of it because it will take care of us… It is what can prevent us from sickness.* Male, Interview participant, Peri-urban (Kokona)

Respondents saw both care and repair as part of a package of overall good net maintenance, although they spoke at greater length about repair than about care practices. Motivations to care for and repair nets most commonly concerned health, citing both overall wellbeing and prevention of malaria and mosquito bites specifically. Another slightly less common motivator was to save money on health expenses. The desire to increase the longevity of nets and comfort while sleeping also came up in a minority of responses. Social norms around housekeeping were also discussed: when asked what they thought when seeing a torn net hanging over a person’s sleeping space, respondents expressed negativity about a person who might have such a net hung over their bed, doubting the person’s cleanliness, hygiene, character, and ability to care for their family.

*If I saw a torn [net] on my sister[‘s] bed, I would think she is careless about her family's health.* Male, Interview participant, Rural (Kokona)

*They are dirty people they don't take care of their net, if you do not take care of your net it will not look good.* Female with child aged under five years, Interview participant, Peri-urban (Kokona)

### Barriers to care and repair of nets

When asked about reasons people in their community may not care for or repair their nets, participant responses fell into six different dimensions: the extent of damage to the net, the cost of repairing, the time required to care and repair, a lack of knowledge on how or why to care and repair, the lack of appreciation for a net, and the preference for a new net. Respondents often reported that they may not know how to care for and repair for nets, and in other cases, the number of holes was the determining factor for whether a net should be repaired; nets with too many holes were perceived to be no longer useful, making net repair not worthwhile. All other reported barriers to net care and repair were linked to the net users’ valuation of the net and of the protection it offered *versus* the cost and time to repair it.

*Because they may not attach any importance to the net… Some people are just carefree. They don't see any necessity in making their nets look good.* Male, Interview participant, Rural (Toto)

A common theme among responses was that while some people may say they do not have the time or money to care for and repair nets, a net owner will make time and money available for repairs and care if they believe it is important to do so.

*There are some people who do not have time but if you think of your health you will have time to repair it.* Female, Focus group participant, Rural (Kokona)

Only extremely poor families (i e, those struggling to find daily food) and individuals who work long hours, such as in agriculture work, were considered exceptions to the expectation that one should prioritize and make time for net care and repair. A final barrier to net repair was the preference among respondents for a new net rather than fixing an old one; respondents much preferred a new net than to repair a torn one, although they also mentioned that the cost of a new net is prohibitive.

*Buying a new one is more expensive…Yes but it is better to get new mosquito net… because repair and re-mending will give stress and take much time, so new one is better.* Male, Focus group participant, Rural (Toto)

### Information sources for net care and repair messaging

Participants were asked to share their ideas for how people in their community can be motivated to take better care of their nets. Suggested methods of sharing net care and repair information with the community included outreach activities, community gatherings or house visits. Health personnel were considered highly trusted sources of information. It was also suggested, to a lesser extent, that local authorities, specifically community leaders, kings, district heads and village heads, could discuss caring for bed nets with community groups, and that churches, mosques and other religious centres could be appropriate fora. Mass media for net care and repair messages was recommended on the basis that it is a commonly used platform for malaria and health messaging and because these platforms, especially radio, have ample reach. Two respondents also suggested including instructions on how to use and care for nets on net labels.

## Discussion

There is little research reported on the behaviour of LLIN users related to net damage and net care and repair. This qualitative study provides novel and valuable insights on the perceptions and attitudes of LLIN users in Nasarawa, Nigeria on the durability of nets, how to extend net longevity and for what reasons. The data have important implications for BCC; if net care and repair behaviour is considered important to net durability, this study elucidates the underpinnings of net care and repair messaging that can be incorporated into communication strategies of LLIN distribution and net use campaigns. These findings can inform future studies on this important behavioural topic, as well as the promotion of net care and repair behaviour in malaria-endemic areas using BCC. The BCC materials developed as a result of this research are available online [24].

Mosquito net users in Nasarawa, Nigeria were knowledgeable about the causes of damage to their nets and ways to mitigate that damage. Participants listed several causes and specific examples of how nets can be damaged in their homes, indicating they have robust experience with net use and net damage. It is noteworthy that, despite the variety of characteristics of sleeping spaces that can cause damage to nets, there was consensus among respondents that the major cause of damage to nets is primarily children, followed by rodents and everyday handling that is not gentle. These findings correlate closely with those of a recent study characterizing the types of damage to 526 nets removed from households in five countries, which found that the most common types of damage to nets are mechanical and rodent damage [25]. These findings allow for communication to focus directly on these issues, and the concept of gentle handling can span multiple types of sleeping spaces. For example BCC about storing nets away from the reach of children during the day and discouraging children playing with or near nets can be messages that are focused on causes of damage and may be applicable to many settings.

Net users in Nasarawa, Nigeria link many of their net care practices to mitigation of net damage. Before this study, there has been little examination of the net care perceptions and practices of net users. In this context, net care primarily involves folding away nets when not in use to avoid damage from children and preventing soiling with food residue to avoid damage from rats. In addition, washing nets when they are perceived to be ‘dirty’ is a central aspect of net care in this setting; however, the washing frequency of nets varied greatly among respondents, with some reporting washing their nets as often as weekly. Washing nets more frequently than every few months, may compromise the protective lifespan of nets. These responses highlight the need for messaging to reduce misperceptions about how often to wash nets and reinforce the use of appropriate products, such as mild soap and water, as opposed to harsh detergents or insecticides. In this study setting, where the vast majority of existing nets are LLINs, there is also confusion about whether insecticide retreatment of nets is necessary; such practice may place unnecessary physical stress on nets depending on how the net is handled, however re-treatment practices were not the focus of this study and require further investigation.

Other important damage mitigation behaviour, such as immediately sewing up holes at the first sign of damage to keep them from getting larger, and daily inspections of net condition, were less mentioned. These are potentially important actions for keeping nets intact, and the fact that they were mentioned much less frequently indicates that communication efforts could boost awareness of and motivation for these actions. In general, messages should focus on making net care and repair behaviour part of the daily household routine; such as stressing proper daily storage of nets.

Repairing a net was universally considered something that net users *should* do and the responsibility of adults in the household. Net repairs were feasible by sewing, patching or tying a knot. Interestingly, net users expressed a preference for repairs that would maintain the visual aesthetic of a new net. Although most participants spoke favourably of repairing nets and expressed high self-efficacy to repair nets, past experience with net repair seems to be limited among this study population, with only 18% of respondents reporting ever repairing a net. This discrepancy between what net users say they should or would do and what they report they actually do may be indicative of the low priority of net repair among other housekeeping behaviour, and communication interventions can specifically address this.

Communication campaigns can benefit from identifying the motivating and hindering factors that may influence health behaviour. This study found that certain social, emotional and personal factors motivate net care and repair behaviour. Net users in Nasarawa are motivated to take care of and repair their nets by the notions of being a ‘good’ parent, caring for and promoting health in their family, avoiding mosquito bites while sleeping, and maintaining the positive opinion of others by keeping a clean and intact net; a person with a damaged and dirty net was perceived as careless and unhygienic in this community. Social norms and the social pressure to maintain the good opinion of others influences net aesthetics and maintenance practices. Therefore, messages can target both health impacts (a well-maintained net will protect the family and community from malaria) as well as the social norm benefits (a well-maintained net will indicate cleanliness and ability to care for one’s family). A final motivating factor that was gleaned from this study is the potential relationship between care and repair of nets and household finances. Net users in Nasarawa, Nigeria believed that keeping a net intact, and therefore and in use, can reduce malaria episodes in the family and prevent the cost of seeking care, taking medicines and being absent from work or school. The notion of saving money by executing care and repair behaviour can be powerful motivators in communication, especially when targeting heads of households.

The preference of some respondents to obtain new nets rather than repairing damaged nets suggests that communication messaging should pay attention to local net replacement options, so that appropriate net replacement recommendations can be given. At the time of this research, cost and limited availability of nets made procuring a new net difficult in that local context. Even when nets may be available and affordable, supply may be limited or unreliable. Thus, it remains important to motivate net users to care for their nets. In the context where replacement nets are reliably and consistently available and affordable, communication to promote seeking and obtaining new LLINs when the household deems their nets are beyond repair can also be important [8].

Barriers to behaviour are key important considerations for health messaging. In this study respondents stated that the cost or availability of needles, thread or patches were not major barriers to net repair for most families, although this should be further explored in other contexts. More important barriers were the time that it takes to repair a net *versus* the perceived benefit of the repair, the difficulty of the repair and the grade of damage to the net. Messages aimed at preventing holes and encouraging the repair of small holes immediately will be important aspects of overcoming these barriers. Yet the largest barrier seems to be in attitudes toward care and repair; many respondents stated that they would make time and money available for care and repair if they believed it was important to do so. Therefore, there is a clear role for BCC to effect changes in attitudes such that net repair becomes priority behaviour.

### Limitations

This study was conducted in two LGAs in one state in Nigeria. It is therefore difficult to conclude that the findings are applicable to other areas or regions in Nigeria, of Africa, or to all net users. As with most qualitative research, there is the possibility of a social desirability bias, given that respondents may wish to present their behaviour regarding net care and repair in a favourable light. On some occasions the data collection team were mistakenly perceived as health workers because health workers frequently distribute nets in the community, and respondents might have tailored their responses based upon the misconception that their responses would have a bearing on receipt of another net. Another limitation to this qualitative study is that LLIN users in Nasarawa may be facing net durability issues for the first time and therefore may have limited experience to discuss. The first campaign to distribute LLINs targeting the general population took place in Nasarawa two years before this study. Prior to this, net distribution had been limited to pregnant women and young children.

## Conclusion

BCC interventions for malaria prevention typically include a component to promote net use, and may include net care messaging, but do not usually include messages on how and why to repair nets. This study revealed nuances about net users’ perceptions on sources of net damage, net care and repair behaviour and the motivating factors and barriers that influence these practices. These findings demonstrate that net care and repair BCC needs to use clear and compelling messages, to improve attitudes and efficacy of net owners to move from knowing they should take care of and repair nets to incorporating this practice into their everyday household routine. At a time when international resources for LLIN campaigns are decreasing, it will become increasingly important to promote actions, such as net care and repair, in future net distributions and routine malaria-prevention communication which may potentially delay the deterioration of LLINs in households.

## Abbreviations

LLIN: Long-lasting insecticidal net
MOH: Ministry of Health
LGA: Local government area
FGD: Focus group discussion
IDI: In-depth interview
BCC: Behaviour-change communication.
